# Supplementary material for: A fault-intrusion-tolerant system and deadline-aware algorithm for scheduling scientific workflow in the cloud
Source: PeerJ Comput Sci. 2021 Nov 2;7:e747. doi: 10.7717/peerj-cs.747 (PMC8576554; doi:10.7717/peerj-cs.747)
Supplement: Supplemental Information 6 [file peerj-cs-07-747-s006.zip › Myproject1.1/docs/org/cloudbus/cloudsim/util/MathUtil.html]

MathUtil


JavaScript is disabled on your browser.


- Overview
- Package
- Class
- Use
- Tree
- Deprecated
- Index
- Help

- Prev Class
- Next Class

- Frames
- No Frames

- All Classes

- Summary:
- Nested |
- Field |
- Constr |
- Method

- Detail:
- Field |
- Constr |
- Method


org.cloudbus.cloudsim.util

## Class MathUtil

- java.lang.Object
- - org.cloudbus.cloudsim.util.MathUtil

- ---

    

  ```
  public class MathUtil
  extends java.lang.Object
  ```

  A class containing multiple convenient math functions.

  Since:
  :   CloudSim Toolkit 3.0

- - ### Constructor Summary

    Constructors

    Constructor and Description

    `MathUtil()`
  - ### Method Summary

    Methods

    | Modifier and Type | Method and Description |
    | --- | --- |
    | `static double[]` | `abs(double[] data)` Abs. |
    | `static int` | `countNonZeroBeginning(double[] data)` Count non zero beginning of the data. |
    | `static int` | `countShortestRow(double[][] data)` Count shortest row. |
    | `static org.apache.commons.math3.stat.regression.OLSMultipleLinearRegression` | `createLinearRegression(double[][] x, double[] y)` |
    | `static org.apache.commons.math3.stat.regression.SimpleRegression` | `createLinearRegression(double[] x, double[] y)` |
    | `static org.apache.commons.math3.stat.regression.SimpleRegression` | `createWeigthedLinearRegression(double[] x, double[] y, double[] weigths)` |
    | `static double[]` | `getLoessParameterEstimates(double[] y)` Gets the loess parameter estimates. |
    | `static double[]` | `getRobustLoessParameterEstimates(double[] y)` Gets the robust loess parameter estimates. |
    | `static org.apache.commons.math3.stat.descriptive.DescriptiveStatistics` | `getStatistics(double[] list)` Returns descriptive statistics for the array of numbers. |
    | `static org.apache.commons.math3.stat.descriptive.DescriptiveStatistics` | `getStatistics(java.util.List<java.lang.Double> list)` Returns descriptive statistics for the list of numbers. |
    | `static double[]` | `getTricubeBisquareWeigts(double[] residuals)` Gets the tricube bisquare weigts. |
    | `static double[]` | `getTricubeWeigts(int n)` Gets the tricube weigts. |
    | `static double` | `iqr(double[] data)` Gets the IQR. |
    | `static double[]` | `listToArray(java.util.List<? extends java.lang.Number> list)` List to array. |
    | `static double` | `mad(double[] data)` Gets the mad. |
    | `static double` | `mean(java.util.List<java.lang.Double> list)` Gets the average. |
    | `static double` | `median(double[] list)` Gets the median. |
    | `static double` | `median(java.util.List<java.lang.Double> list)` Gets the median. |
    | `static double` | `stDev(java.util.List<java.lang.Double> list)` Gets the standard deviation. |
    | `static double` | `sum(java.util.List<? extends java.lang.Number> list)` Sums a list of numbers. |
    | `static double[]` | `trimZeroTail(double[] data)` Trim zero tail. |
    | `static double` | `variance(java.util.List<java.lang.Double> list)` Variance. |

    - ### Methods inherited from class java.lang.Object

      `clone, equals, finalize, getClass, hashCode, notify, notifyAll, toString, wait, wait, wait`

- - ### Constructor Detail


    - #### MathUtil

      ```
      public MathUtil()
      ```
  - ### Method Detail


    - #### sum

      ```
      public static double sum(java.util.List<? extends java.lang.Number> list)
      ```

      Sums a list of numbers.

      Parameters:
      :   `list` - the list

      Returns:
      :   the double


    - #### listToArray

      ```
      public static double[] listToArray(java.util.List<? extends java.lang.Number> list)
      ```

      List to array.

      Parameters:
      :   `list` - the list

      Returns:
      :   the double[]


    - #### median

      ```
      public static double median(java.util.List<java.lang.Double> list)
      ```

      Gets the median.

      Parameters:
      :   `list` - the list

      Returns:
      :   the median


    - #### median

      ```
      public static double median(double[] list)
      ```

      Gets the median.

      Parameters:
      :   `list` - the list

      Returns:
      :   the median


    - #### getStatistics

      ```
      public static org.apache.commons.math3.stat.descriptive.DescriptiveStatistics getStatistics(java.util.List<java.lang.Double> list)
      ```

      Returns descriptive statistics for the list of numbers.

      Parameters:
      :   `list` - - the list of numbers. Must not be null.

      Returns:
      :   - descriptive statistics for the list of numbers.


    - #### getStatistics

      ```
      public static org.apache.commons.math3.stat.descriptive.DescriptiveStatistics getStatistics(double[] list)
      ```

      Returns descriptive statistics for the array of numbers.

      Parameters:
      :   `list` - - the array of numbers. Must not be null.

      Returns:
      :   - descriptive statistics for the array of numbers.


    - #### mean

      ```
      public static double mean(java.util.List<java.lang.Double> list)
      ```

      Gets the average.

      Parameters:
      :   `list` - the list

      Returns:
      :   the average


    - #### variance

      ```
      public static double variance(java.util.List<java.lang.Double> list)
      ```

      Variance.

      Parameters:
      :   `list` - the list

      Returns:
      :   the double


    - #### stDev

      ```
      public static double stDev(java.util.List<java.lang.Double> list)
      ```

      Gets the standard deviation.

      Parameters:
      :   `list` - the list

      Returns:
      :   the double


    - #### mad

      ```
      public static double mad(double[] data)
      ```

      Gets the mad.

      Parameters:
      :   `data` - the data

      Returns:
      :   the mad


    - #### iqr

      ```
      public static double iqr(double[] data)
      ```

      Gets the IQR.

      Parameters:
      :   `data` - the data

      Returns:
      :   the IQR


    - #### countNonZeroBeginning

      ```
      public static int countNonZeroBeginning(double[] data)
      ```

      Count non zero beginning of the data.

      Parameters:
      :   `data` - the data

      Returns:
      :   the int


    - #### countShortestRow

      ```
      public static int countShortestRow(double[][] data)
      ```

      Count shortest row.

      Parameters:
      :   `data` - the data

      Returns:
      :   the int


    - #### trimZeroTail

      ```
      public static double[] trimZeroTail(double[] data)
      ```

      Trim zero tail.

      Parameters:
      :   `data` - the data

      Returns:
      :   the double[]


    - #### getLoessParameterEstimates

      ```
      public static double[] getLoessParameterEstimates(double[] y)
      ```

      Gets the loess parameter estimates.

      Parameters:
      :   `y` - the y

      Returns:
      :   the loess parameter estimates


    - #### createLinearRegression

      ```
      public static org.apache.commons.math3.stat.regression.SimpleRegression createLinearRegression(double[] x,
                                                                                     double[] y)
      ```


    - #### createLinearRegression

      ```
      public static org.apache.commons.math3.stat.regression.OLSMultipleLinearRegression createLinearRegression(double[][] x,
                                                                                                double[] y)
      ```


    - #### createWeigthedLinearRegression

      ```
      public static org.apache.commons.math3.stat.regression.SimpleRegression createWeigthedLinearRegression(double[] x,
                                                                                             double[] y,
                                                                                             double[] weigths)
      ```


    - #### getRobustLoessParameterEstimates

      ```
      public static double[] getRobustLoessParameterEstimates(double[] y)
      ```

      Gets the robust loess parameter estimates.

      Parameters:
      :   `y` - the y

      Returns:
      :   the robust loess parameter estimates


    - #### getTricubeWeigts

      ```
      public static double[] getTricubeWeigts(int n)
      ```

      Gets the tricube weigts.

      Parameters:
      :   `n` - the n

      Returns:
      :   the tricube weigts


    - #### getTricubeBisquareWeigts

      ```
      public static double[] getTricubeBisquareWeigts(double[] residuals)
      ```

      Gets the tricube bisquare weigts.

      Parameters:
      :   `residuals` - the residuals

      Returns:
      :   the tricube bisquare weigts


    - #### abs

      ```
      public static double[] abs(double[] data)
      ```

      Abs.

      Parameters:
      :   `data` - the data

      Returns:
      :   the double[]


- Overview
- Package
- Class
- Use
- Tree
- Deprecated
- Index
- Help

- Prev Class
- Next Class

- Frames
- No Frames

- All Classes

- Summary:
- Nested |
- Field |
- Constr |
- Method

- Detail:
- Field |
- Constr |
- Method
